# Single-center versus multi-center biparametric MRI radiomics approach for clinically significant peripheral zone prostate cancer

**DOI:** 10.1186/s13244-021-01099-y

**Published:** 2021-10-21

**Authors:** Jeroen Bleker, Derya Yakar, Bram van Noort, Dennis Rouw, Igle Jan de Jong, Rudi A. J. O. Dierckx, Thomas C. Kwee, Henkjan Huisman

**Affiliations:** 1 Departments of Radiology, Nuclear Medicine and Molecular Imaging, Medical Imaging Center, University Medical Center Groningen, University of Groningen, Hanzeplein 1, 9700 RB Groningen, The Netherlands; 2 Meditech Building, Room n305, L.J. Zielstraweg 1, 9713 GX Groningen, The Netherlands; 3 Department of Radiology, Martini Hospital Groningen, Van Swietenplein 1, 9728 NT Groningen, The Netherlands; 4 Department of Urology, University Medical Center Groningen, University of Groningen, Hanzeplein 1, 9700 RB Groningen, The Netherlands; 5 Department of Radiology and Nuclear Medicine, Radboud University Medical Center, Geert Grooteplein Zuid 10, 6525 GA Nijmegen, The Netherlands

**Keywords:** MRI, Prostatic neoplasms, Supervised machine learning

## Abstract

**Objectives:**

To investigate a previously developed radiomics-based biparametric magnetic resonance imaging (bpMRI) approach for discrimination of clinically significant peripheral zone prostate cancer (PZ csPCa) using multi-center, multi-vendor (McMv) and single-center, single-vendor (ScSv) datasets.

**Methods:**

This study’s starting point was a previously developed ScSv algorithm for PZ csPCa whose performance was demonstrated in a single-center dataset. A McMv dataset was collected, and 262 PZ PCa lesions (9 centers, 2 vendors) were selected to identically develop a multi-center algorithm. The single-center algorithm was then applied to the multi-center dataset (single–multi-validation), and the McMv algorithm was applied to both the multi-center dataset (multi–multi-validation) and the previously used single-center dataset (multi–single-validation). The areas under the curve (AUCs) of the validations were compared using bootstrapping.

**Results:**

Previously the single–single validation achieved an AUC of 0.82 (95% CI 0.71–0.92), a significant performance reduction of 27.2% compared to the single–multi-validation AUC of 0.59 (95% CI 0.51–0.68). The new multi-center model achieved a multi–multi-validation AUC of 0.75 (95% CI 0.64–0.84). Compared to the multi–single-validation AUC of 0.66 (95% CI 0.56–0.75), the performance did not decrease significantly (*p* value: 0.114). Bootstrapped comparison showed similar single-center performances and a significantly different multi-center performance (*p* values: 0.03, 0.012).

**Conclusions:**

A single-center trained radiomics-based bpMRI model does not generalize to multi-center data. Multi-center trained radiomics-based bpMRI models do generalize, have equal single-center performance and perform better on multi-center data.

**Supplementary Information:**

The online version contains supplementary material available at 10.1186/s13244-021-01099-y.

## Key points

- Multi-center radiomics-based bpMRI models generalize to new multi-center and single-center data.
- A single-center, single-vendor radiomics-based bpMRI model lacks multi-center generalization.
- Multi-center developed models match the single-center validation of a single-center model.
- Multi-center developed models outperform single-center models in a multi-center validation.

## Introduction

In 2020, prostate cancer (PCa) is expected to be the most common cancer with the second highest mortality rate among western males [1]. Multiparametric magnetic resonance imaging (mpMRI) has led to an increase in diagnostic performance for clinically significant (CS) PCa [2]. However, diagnostic performance remains suboptimal [3] and extensive radiologic experience is required to achieve passable csPCa accuracy [4].

Many recent efforts to improve diagnostic performance for csPCa have used some form of artificial intelligence (AI) [5]. Among these studies, however, there is a lack of proper external validation [6]. The vast majority developed their models on single-center, single-vendor (ScSv) datasets making them potentially prone to both center and MRI dependencies. For most AI approaches, for example, feature-based techniques (e.g., radiomics [7]), differences in center, vendor or protocol might affect MRI voxel intensity ranges and in turn influence generalization [8]. Although steps have been taken to standardize features [9], no studies have investigated what occurs during an external multi-center, multi-vendor (McMv) validation of a ScSv model.

In a previous study, it was shown that a radiomics-based biparametric MRI (bpMRI) approach has diagnostic potential in discriminating csPCa from non-CS PCa in the peripheral zone (PZ) [10]. In that previous study, a selection of the publicly available ScSv challenge dataset, (*n* = 262, only PZ lesions from ProstateX) [11] was used for training and testing. This approach was in line with other published radiomics studies at that time, all of them based on ScSv datasets [6]. With radiomics, the user tries to quantify tumor imaging data by extracting a diverse range of statistical and texture-based features. After extraction, the goal is to look for relevant relations between these features and the lesion labels (CS PCa vs. non-CS PCA), i.e., supervised learning. The relevant features are then supplied to a model with the aim of quantifying tumor phenotype, ideally in such a way that CS PCa predictions on new never seen before MRI PCa data should become possible. However, in practice good generalization is one of the biggest challenges of radiomics [12, 13]. The extent of center and machine dependencies of popular single-center developed models and thus their behavior on new external data remains unclear. We hypothesize that single-center model performance will degrade when used on McMv data even when trained on multi-center data.

In this study, we investigate the diagnostic performance of a previously developed csPCa radiomics approach trained and validated with a mix of a ScSv or McMv datasets.

## Materials and methods

### Patient data

Data collection was approved by the institutional review board of each of the main contributing medical centers approached for retrospective collection University Medical Center Groningen (UMCG, Groningen, The Netherlands) and Martini Hospital Groningen (Groningen, The Netherlands)). Informed consent was waived due to the retrospective nature of the study. Because of clinical suspicion of PCa, a group of 930 patients, which consisted almost exclusively of white European males, was investigated between 2014 and 2020 using a combination of MRI (9 hospitals, 2 vendors, 8 types; Additional file 1) and systematic or targeted biopsy techniques (after MRI, maximum period 1 year). MRI studies followed protocol recommendations found in the prostate imaging-reporting and data system guidelines [14]; therefore, protocol settings (such as *b*-values and corresponding ADC maps) did not differ largely (Protocol settings: Additional file 1).

Among these 930 patients, a total of 1151 lesions were assigned a PI-RADS score (1–5) by two experienced uroradiologists (D.Y. 8 years, D.R. 10 years). Approximately half of the 1151 lesions were graded independently by one uroradiologist, and the remaining half was graded independently by the other uroradiologist. Prostate MRI was read according to the PIRADS v2 guidelines [15], and each radiologist was blinded for pathological results and clinical follow-up. DWI and TW2 images were analyzed for the detection of suspicious lesions. For PZ, DWI is the primary determining sequence. However, findings were always correlated with the T2W images. Due to independent grading and no overlap between lesion groups, no consensus decisions had to be made.

A total of 641 lesions were biopsy naïve, 304 lesions had previously been negative on transrectal ultrasound biopsy (TRUS) biopsies, and 206 had previously been positive on TRUS biopsy. PI-RADS 3–5 lesions were subsequently managed by either non-targeted TRUS biopsy, targeted MRI-TRUS fusion, targeted cognitive TRUS fusion, prostatectomy or in-bore MR guided. All biopsies after MRI occurred in the two main contributing centers University Medical Center Groningen (UMCG, Groningen, The Netherlands) and Martini Hospital Groningen (Groningen, The Netherlands), distribution see Table 1). Following biopsy, pathology assigned international society of urological pathology (ISUP) grades [16] were used as reference standard. ISUP grade ≥ 2 was regarded as CS lesions, while lesions with a ISUP grade of 1 were considered nonsignificant (non-CS) entities. Overview of scanner type and manufacturer can be found in Additional file 1.

**Table 1:** Targeted biopsy distribution for the final selection of 335 multi-center lesions

Biopsy technique	MRI-TRUS fusion	Cognitive fusion	Prostatectomy	In bore MR Guided
Occurrence	296	24	11	4
Location	Hospital A: 177 Hospital B: 119	Hospital B	Hospital B	Hospital B

Only hospitals A and B are represented in the biopsy distribution since the 7 other centers are referring institutions which do not perform biopsies. Instead, patients receive MRI scans in one of these 7 centers and are then referred to hospitals A or B for MRI-TRUS fusion if needed

### Radiomics-based bpMRI approach

**Fig. 1:**
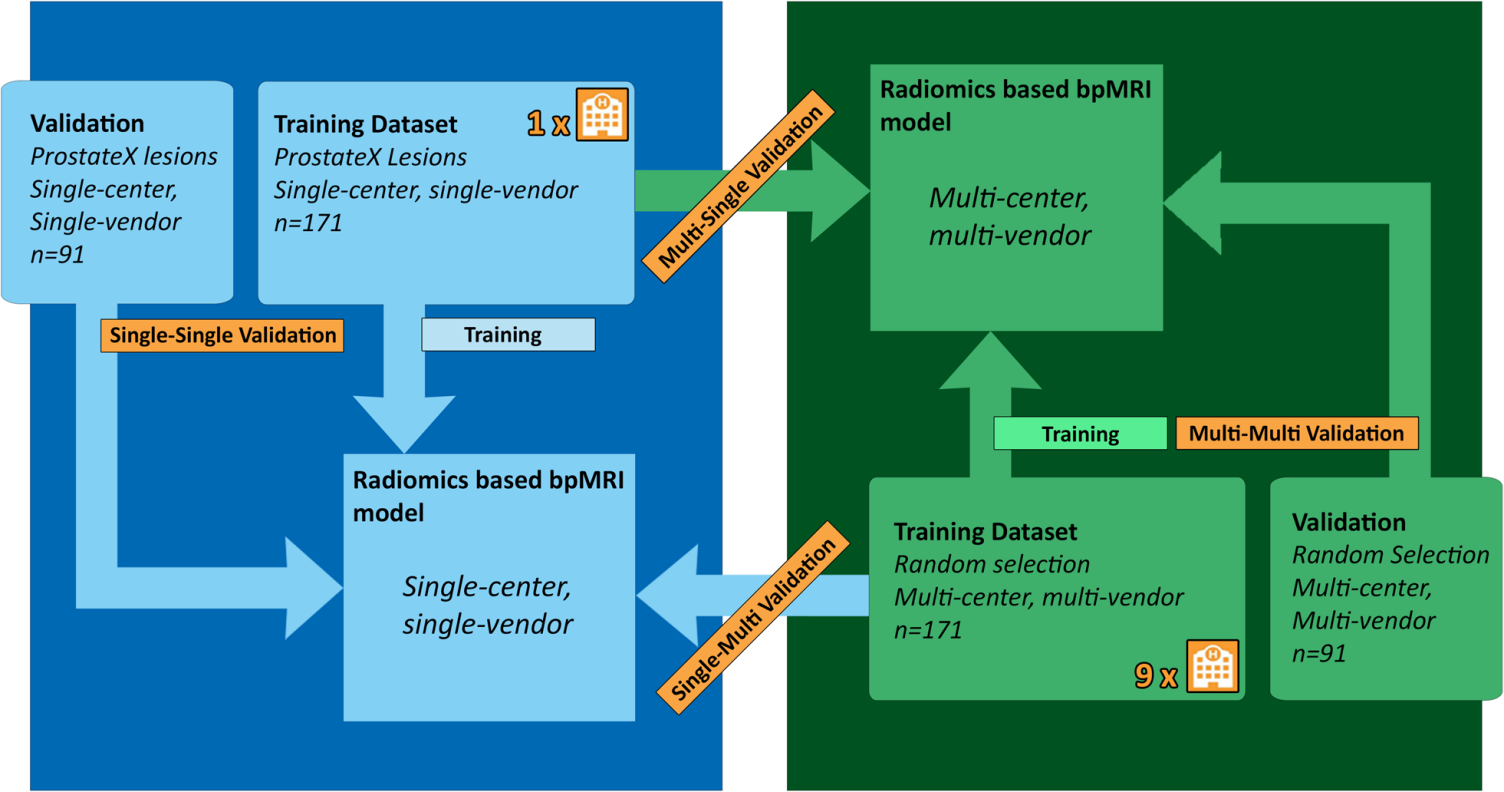
Validation overview with the development datasets and each of the validations described. Single–single validation = single-center, single-vendor model validated on single-center, single-vendor data. Multi–multi-validation = multi-center, multi-vendor model validated on multi-center, multi-vendor data. In the multi–single and single–multi-combinations, the first word refers to the model, while the second refers to the dataset

The previously developed radiomics-based bpMRI PZ csPCa approach [10] was based on T2-weighted and diffusion-weighted imaging (DWI, *b*-values: 800 and 1400 s/mm^2^) and the apparent diffusion coefficient map (ADC map). Inclusion of dynamic contrast enhanced imaging features did not significantly increase performance and was omitted to save time and reduce costs [10]. Development occurred on the publicly available single-center, single-vendor ProstateX challenge dataset consisting of prospectively graded PI-RADS 3–5 lesions [11]. ProstateX dataset patients were scanned at the Radboud University Medical Center (Nijmegen, The Netherlands) in 2012 on a 3T MRI scanner (Siemens Healthcare). ProstateX patients had a median age of 66 (range 48–83 years) with a median PSA level of 13 ng/ml (range 1–56 ng/ml). Two hundred and six patients with 262 lesions were used for approach development, split in a training dataset of 171 lesions and a test dataset of 91 lesions. A total of 564 features were extracted for each lesion using an auto-fixed volume of interest (VOI). The features belonged to the categories: first-order statistics, gray-level co-occurrence matrix, gray-level run length matrix, gray-level size zone matrix, neighboring gray tone difference matrix or gray-level dependence matrix. Addition of extra image filters was omitted since the filters did not lead to an improvement for the previously developed approach [10]. The auto-fixed VOI is a semi-automatic segmentation technique where an operator defined the most aggressive place in a lesion, i.e., the lesion voxel with lowest ADC value. Around this voxel, a 12-mm spherical VOI was constructed and used for VOI-based radiomics feature extraction. A bpMRI example of this technique can be found in Additional file 1. ProstateX lesions coordinates were previously checked by an expert radiologist (D.Y. 8 years, blinded for pathological results and clinical follow-up) and where needed adjusted to ensure an appropriate location in the tumor [17]. After feature extraction with PyRadiomics, features were discretized [9] and the most relevant features were selected using joint mutual information maximization multivariate feature selection [18]. Relevant features were supplied to an extreme gradient boosting algorithm [19], and model hyperparameters were optimized. The best performing ScSv model in the previous study achieved a single-center validation (single–single validation) area under the curve (AUC) of 0.816 (95% CI 0.710–0.920). Figure 1 gives an overview of the datasets used for development and validation.

### Data selection

**Fig. 2:**
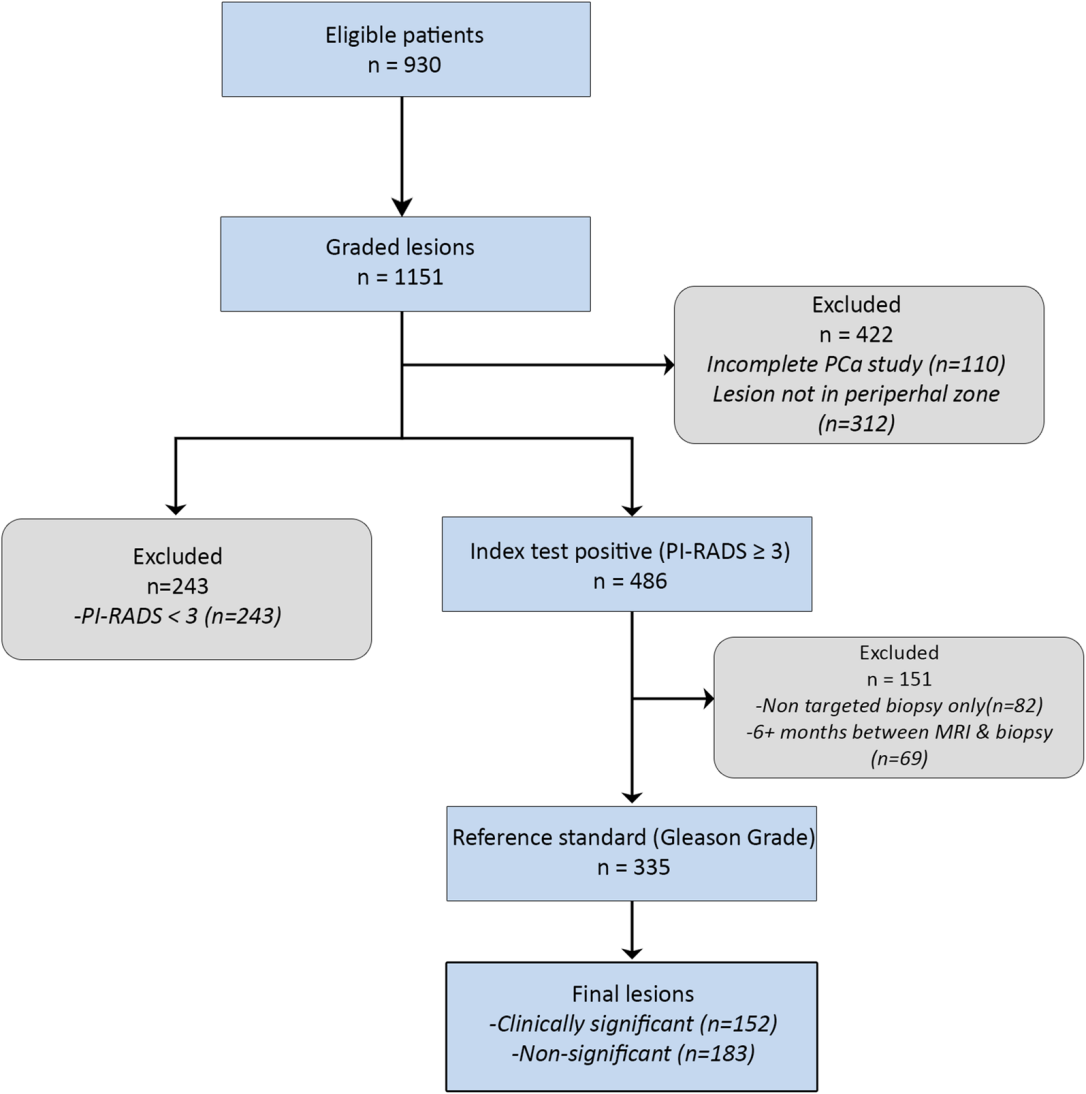
STARD flowchart

To allow single–multi-validation (i.e., McMv validation of the ScSv model that resulted from the radiomics-based bpMRI PZ csPCa approach), multiple data selection steps were required to emulate the structure and parameters of the ProstateX dataset (Fig. 2). Lesions with PI-RADS scores lower than 3 were excluded since these are not biopsied in clinical practice and the original development dataset (ProstateX) also consisted of prospectively graded PI-RADS 3–5 lesions. The set of features used by the model required MRI examinations to include T2-weighted and DWI (*b*-values 800, 1400 s/mm^2^) sequences, and an ADC map. Finally, the lesions had to be located in the PZ of the prostate and ISUP grade had to be based on a spatially matched pathology specimen (i.e., MRI-TRUS fusion, targeted cognitive TRUS fusion, prostatectomy or in-bore MR guided biopsy) taken within 6 months of the MRI study.

### Multi-center, multi-vendor validation of a single-center, single-vendor model

Following data selection steps described in Fig. 2 a group of McMv lesions remained that met model use conditions for comparability. To ensure proper unbiased comparison between the validations of the single-center and the new multi-center model, a selection of 262 lesions was taken at random while preserving the dataset distribution. To adhere to the radiomics-based bpMRI PZ csPCa approach and ensure validation comparison (i.e., create the same training and test distribution as the ProstateX dataset), this group was further divided into a training dataset with 171 lesions and a validation set of 91 lesions. Besides the role as a training dataset, these 171 lesions were used in single–multi-validation and directly fed to the previously trained ScSv model and performance metrics (sensitivity, specificity, receiver operating characteristic (ROC) analysis and corresponding AUCs) were calculated. Detailed overview can be found in Fig. 1.

### Development and validation of a multi-center, multi-vendor model

The previously developed radiomics-based bpMRI PZ csPCa approach [10] was used for the development of a McMv-based model. As mentioned previously, the development dataset of 262 McMv lesions was already created and split (*n* = 262, training *n* = 171, validation = 91) [10]. A total of 564 multi-center radiomics features (feature categories mentioned in radiomics-based bpMRI approach paragraph) were extracted using the same semi-automatic “auto-fixed” 12 mm VOI strategy previously developed as part of the bpMRI PZ csPCa approach [10]. A single experienced uroradiologist (D.Y. 8 years) retrospectively indicated the lesion area in which the voxel with the lowest ADC value was selected (i.e., lesion pinpoint). The radiologist was blinded to all clinical information including pathology results. The training dataset was used for the development of the new McMv model precisely following the bpMRI PZ csPCa approach. Instead of the previous randomized feature selection and hyperparameter optimization, Bayesian optimization was preferred for feature selection and hyperparameter optimization due to its computing speed and reproducible and unbiased nature [20]. The method for feature selection remained the filter-based Joint mutual information maximization which was found to be the most successful during the development of the bpMRI PZ csPCa approach [10, 18]. Bayesian optimization was implemented as a sequential model-based optimization through Optuna [21] and can be easily reproduced due to being an automated hyperparameter optimization framework. By creating a nested cross-validation for JMIM inside the Optuna model optimization and selecting features that occur in each randomized fold, the feature selection should be reproducible and robust to different compositions of multi-center data. Multi–multi-validation was performed by validating the resulting McMv model on the separate 91 multi-center lesions. Subsequently, multi–single-validation was performed by validating the new model on 171 single-center lesions that were used for initial ScSv radiomics-based bpMRI model development (ProstateX, training dataset [10]). This provided an equal number of lesions for the total development and validation datasets (*n* = 171 and *n* = 91) for both models, allowing for unbiased validation comparisons.

### Statistical analysis

95% confidence intervals for all performance metrics were created using 5000 times bootstrapping the model test results. Sensitivity, specificity, AUC values and ROC curves were acquired for all validations. Youden’s index was used to find the best cut-off value for sensitivity and specificity. The ROC curves for all validations were compared creating a total of four comparisons. Due to multiple comparisons, Bonferroni correction was applied creating a new *p* value limit of 0.0125. Both comparisons were calculated using 5000 times ROC bootstrapping. All analyses were performed using R (Version 4.0.3 Copyright (C) 2020 The R Foundation for Statistical Computing) with the pROC package [22].

## Results

### Multi-center dataset characteristics

From the total dataset of 1151 McMv lesions, 335 lesions remained that met data selection criteria (Fig. 2). A total of 236 lesions were scanned in either Hospital A or Hospital B, while the remaining 99 lesions were scanned in seven smaller regional medical centers. The biopsy technique distribution for the 335 eligible lesions is given in Table 1. The final set included 79 PI-RADS 3 lesions, 219 PI-RADS 4 lesions and 30 PI-RADS 5 lesions. Of the final dataset 94 lesions had a negative targeted biopsy after MRI, 89 lesions were ISUP 1, another 98 were ISUP 2, 31 were ISUP 3, 11 ISUP 4 and 12 ISUP 5. 

From the 335 eligible McMv lesions, 262 McMv lesions were randomly selected while preserving dataset distribution. The group of 262 PZ PCa lesions consisted of 113 CS lesions and 149 non-CS entities. Patient age ranged from 60 to 87 years with a median of 69 years, while PSA levels ranged from 0.79 to 34 µg/L with a median of 7.9 µg/L. To match previous development, the lesions were split in a training set of 171 lesions and a test set of 91 lesions.

### Single–multi-validation

Validation of the ScSv model on the McMv lesions led to a sensitivity of 0.30 (95% CI 0.20–0.40) with a specificity of 0.88 (95% CI 0.82–0.95) and an AUC of 0.59 (95% CI 0.51–0.68) for the ability to discriminate CS PCa from non-CS PCa in the PZ using an optimized threshold (Fig. 1, single–multi-validation). Confusion matrix for the single–multi-validation can be found in Table 2. Bootstrapped comparison of the single–multi-validation and previously acquired single–single validation showed a significant performance reduction (*p* value: 0.002). For a better understanding of the single–multi-validation, an overview of the top 25 most important features of a total of 76 model features, for the ScSv model, is given in Fig. 4.

**Table 2:** Confusion matrix for the single–multi-validation

Single–multi-validation (*n* = 171)	Predicted significant PCa	Predicted nonsignificant entity	
Label significant PCa	TN = 71	FP = 21	92
Label nonsignificant entity	FN = 49	TP = 30	79
	120	51	

*PCa* prostate cancer, *TN* true negative, *FP* false positive, *FN* false negative, *TP* true positive

### Multi–multi-validation

The newly developed McMv model achieved a multi–center-validation sensitivity of 0.50 (95% CI 0.35– 0.65), a specificity of 0.88 (95% CI 0.79–0.98) and an AUC of 0.75 (95% CI 0.64–0.84) (Fig. 1, multi–multi-validation). Multi–multi-validation confusion matrix can be found in Table 3.

**Table 3:** Confusion matrix for the multi–multi-validation

Multi–multi-validation (*n* = 91)	Predicted significant PCa	Predicted nonsignificant entity	
Label significant PCa	TN = 33	FP = 15	48
Label nonsignificant entity	FN = 16	TP = 27	43
	49	42	

*PCa* prostate cancer, *TN* true negative, *FP* false positive, *FN* false negative, *TP* true positive

### Multi–single validation

The ScSv validation of the McMv model achieved a sensitivity of 0.37 (95% CI 0.23–0.54), a specificity of 0.90 (95% CI 0.85–0.95) and an AUC of 0.66 (95% CI 0.56–0.75) (Fig. 1, multi–single validation). Confusion matrix for the multi–single validation is shown in Table 4. Bootstrapped comparison of the multi–multi-validation and the multi–single validation did not show a significant difference (*p *value: 0.114). To better understand the differences between a single-center and multi-center radiomics model, another detailed overview of the top 8 McMv model features, of a total of 56 model features, is given in Fig. 5.

**Table 4:** Confusion matrix for the multi–single validation

Multi–single validation (*n* = 171)	Predicted significant PCa	Predicted nonsignificant entity	
Label significant PCa	TN = 120	FP = 16	136
Label nonsignificant entity	FN = 22	TP = 13	35
	142	29	

*PCa* prostate cancer, *TN* true negative, *FP* false positive, *FN* false negative, *TP* true positive

**Fig. 3:**
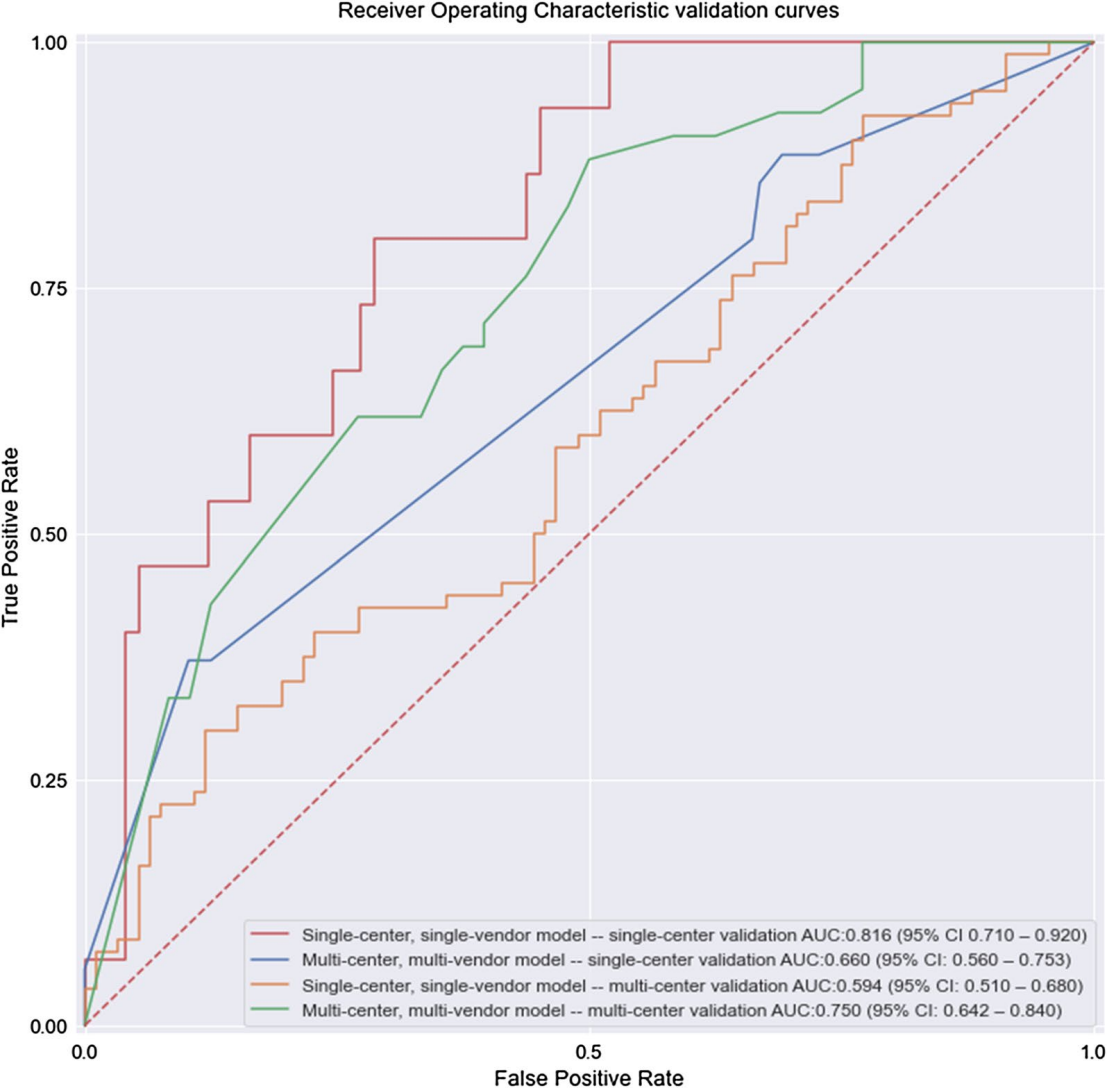
Receiver operating characteristic curves for all training and validation combinations

Bootstrapped comparison of the ROCs for both single-center and multi-center validation results shows that both models have similar single-center performances (*p *value = 0.03). Further ROC bootstrapped comparison showed that the multi-center model had a significantly better multi-center performance (*p *value: 0.012). All validation ROCs (including the previous single–single validation [10]) can be found in Fig. 3 with corresponding AUCs in Table 5.

**Table 5:** AUCs for the test results and external validation of the single-center, single-vendor model and the multi-center, multi-vendor model

	Single-center, single-vendor model	Multi-center, multi-vendor model	*p* value
Single-center validation (AUC)	0.82 (95% CI 0.71–0.92)	0.66 (95% CI 0.56–0.75)	0.03
Multi-center validation (AUC)	0.59 (95% CI 0.51–0.68)	0.75 (95% CI 0.64–0.84)	0.012
Percentage change	− 27.2%	− 12%	x
*p* value	0.002	0.114	

Percentage change refers to the change in performance for the development validation and external validation. For the single-center model, this means the change from single-center validation to multi-center validation, and for the multi-center model this mean the change from multi-center validation to single-center validation

## Discussion

The ScSv radiomics-based bpMRI PCa model suffered from significant performance reduction when used in a McMv setting (− 28.1%, compared to single-center validation AUC: 0.594 vs. 0.816, *p *value: 0.002). The use of a McMv model does not suffer from a significant performance reduction between multi-center and single-center validation (− 12%, AUC: 0.750 vs. 0.660, *p *value: 0.114) and even performs better on multi-center data than the single-center model (AUC 0.750 vs. 0.594, *p *value: 0.012). The nonsignificant reduction between the multi-center and single-center validation of the multi-center model seems to suggest that the use of a McMv dataset improves generalization.

**Fig. 4:**
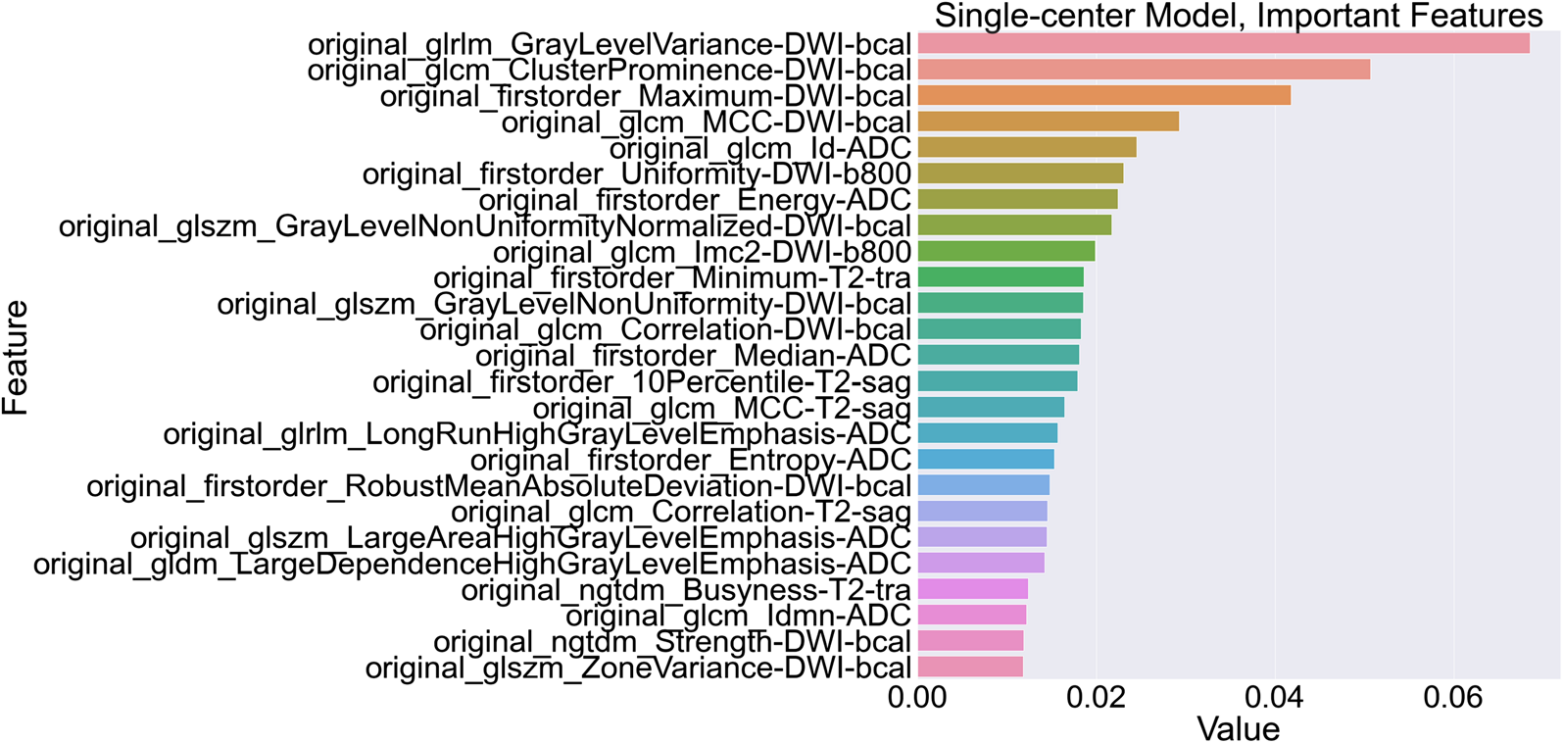
“Gain” values for the top 25 most important features of the single-center model trained on a dataset containing 171 single-center PCa lesions. Higher gain means a higher positive effect on the end performance

**Fig. 5:**
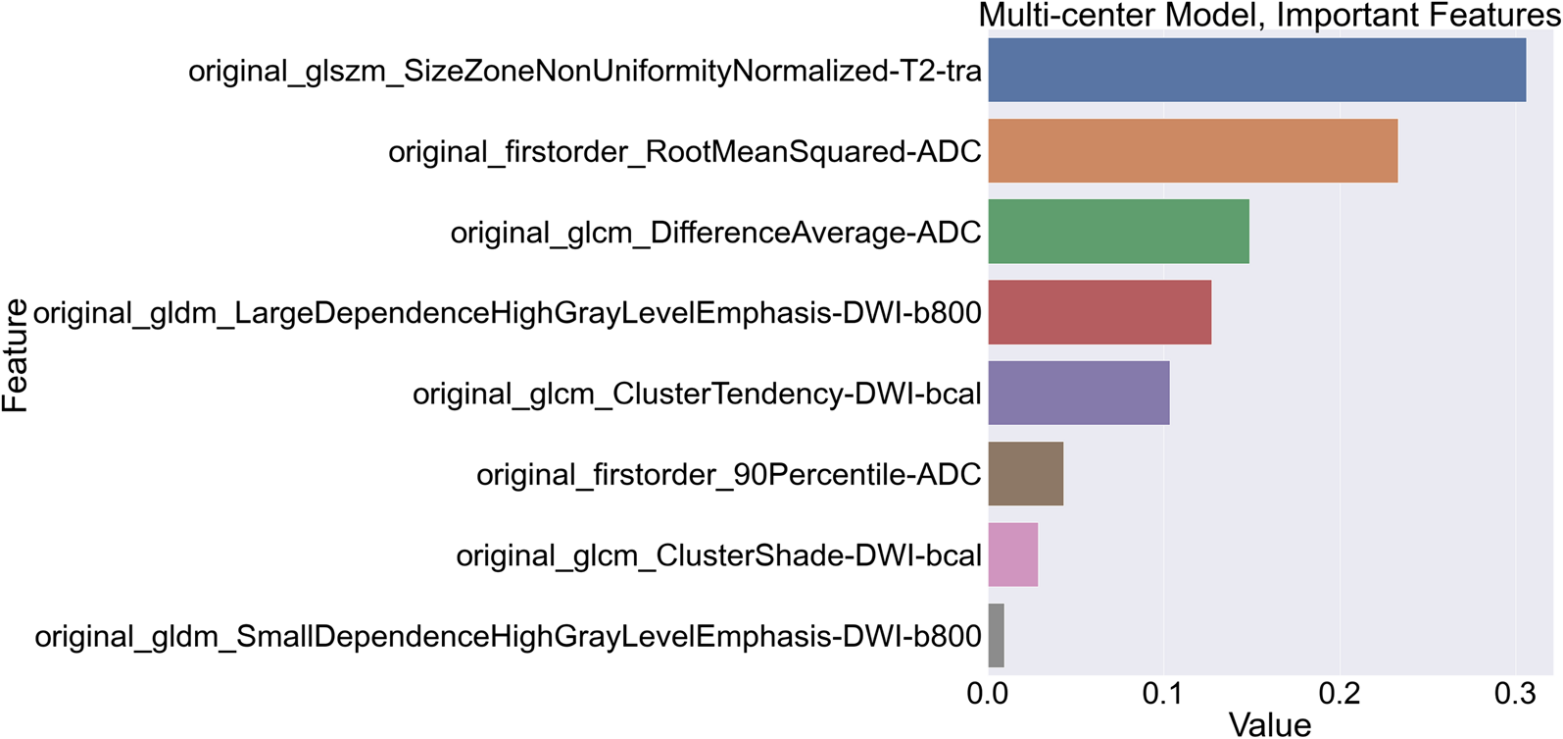
“Gain” values for the top 8 most important features of the multi-center model trained on a dataset containing 171 multi-center PCa lesions. Higher gain means a higher positive effect on the end performance

Interestingly, even though the multi–multi-validation score does not seem all that great the multi-center model has better generalization, suffers from less dependencies, and performs significantly better in a multi-center setting than a single-center development approach. While numerically the single–single validation seems noticeably higher, this is not a significant difference when corrected for multiple comparisons. Though it seems easier to achieve higher scores for the single-center model, this might be explained by the lower complexity of the single-center dataset. Because of the lack of issues such as diverse protocols, larger radiomics feature ranges and different center, vendor and machine dependencies the feature selection can focus on features that heavily correlate with the labels (CS PCa vs. non-CS) and thus increase performance. This is also visible in the large number of features that are found to be important for the final prediction (Fig. 4). Only the top 25 features are given, but all of the 76 model features appear to play a part in the final prediction. Due to the overall homogeneity of the dataset, the features that were selected during cross-validated optimization were not specifically selected for different distributions of the same data. While this does appear to be the case for the multi-center model (Fig. 5) as only 8 out of 56 features appear to affect the final prediction. The other features are not redundant but rather more specified and will not have a large presence in the final gradient boosted trees. A group of 22 features appear in both the McMv and ScSv models, 11 features are extracted from DWI at a *b*-value 1400 s/mm^2^, 8 features originate from the ADC map, and the final 2 features are based on T2-weighted imaging. Interestingly, almost all of these 22 features are either first-order or gray-level co-occurrence features which might suggest that these feature types are slightly more robust to changes in dataset distributions. Of the 22 features, only 2 features affect the final prediction of both the McMv and ScSv models; first-order root mean squared of the ADC map and gray-level co-occurrence cluster tendency of DWI at a *b*-value 1400 s/mm^2^. Root mean squared is the square root of the mean of all squared image intensity values and is a measure of the magnitude of image values, while cluster tendency is a measure for the grouping of voxels with similar intensity values. The increased complexity of the multi-center dataset might be another explanation for its seemingly lower performance. Patient studies were created on different machines from different vendors which used a diverse range of MRI settings (Additional file 1). Especially voxel spacing or other protocol settings influencing voxel intensity ranges are expected to affect radiomic feature ranges [23]. Dataset processing, which might solve for multi-center complexity issues [9], was omitted due to the previously developed radiomics-based bpMRI approach homogeneity and a missing standard for bpMRI PCa radiomics. With proper inclusion of these steps, it is expected that both the single-center and multi-center validation performance of the new multi-center model will improve [9, 24]. However, proper implementation requires extensive optimization experiments since just selecting a plausible set of post-processing settings does not necessarily lead to an improvement [25]. Additionally, when looking at the current generalization of the multi-center model it can be speculated that even when omitting these pre-processing steps the multi-center training is able to learn information about the diverse data. Observation of the feature importance scores given in Figs. 4 and 5 might suggest that T2-weighted and ADC features are less sensitive to dependencies than features taken from DWI with a calculated *b*-value although the discrepancy between the total number of model features and the features found important for the multi-center model interferes with drawing any true conclusions.

The number of studies that developed machine learning approaches on single-center datasets for PCa using radiomics is extensive [6]. However, there is a lack of studies that subsequently investigated the performance of their approach or model on McMv data [6]. Only one recent study by Castillo et al. [25] investigated the generalizability of a radiomics model for classifying PCa. Although this study differed from the current one and used a suboptimal post-processing approach, they also found a lacking generalizability for single-center models [25]. The test AUC for their single-center models was lower than ours (0.816 vs. 0.75) and the multi-center model did not manage to improve on this even with preprocessing. Furthermore, no comparisons between the models were performed. A recent study by Bournonne et al. [26] of an MRI-derived radiomics model to predict biochemical recurrence after surgery for high-risk prostate cancer also focused on validation. Although the goal, sequences and development of Bournonne et al.’s model were different than ours, it still used ADC derived radiomic features that even with model re-training failed to achieve a good score on an external validation set. Application of ComBat compensation, a technique, especially developed to compensate feature differences that occur due to different centers, vendors and scanners, did not lead to significant differences. Unfortunately, their model only used one radiomic feature, so true exclusion of ComBat compensation for future preprocessing optimization might be ill-advised, especially when also accounting for the positive effects of ComBat compensation on multi-center MR radiomics features found by Orlhac et al. [27]

Our current study suffers from three main limitations. First, all investigations were performed on PCa lesions in the PZ. While the expectation is that recommendations for multi-center development hold up for all prostate zones, it cannot be excluded that transition zone (TZ) lesions have a different susceptibility to center, vendor or protocol dependencies. Fortunately, the large majority of PCa lesions are located in the PZ; only around 20% can be found in the other zones [28]. However, TZ lesions remain important for future clinical implementation. Future research, which will no longer be limited by previous approach validations and data availability, should include TZ lesions. Second, the inclusion of extra preprocessing besides the current normalization and gray value discretization might have an effect on the generalization for both the ScSv and McMv models. Recently, Ligero et al. have found a significant reduction of computed tomography radiomics feature variability by implementing both interpolation and intensity harmonization (ComBat compensation) [29]. Additionally, Delli Pizzi et al. [30] successfully created an MRI radiomics model that predicted tumor treatment response based on three resampled datasets. However, proper implementation of interpolation and intensity harmonization requires extensive optimization experiments since there is no evidence-based standard for MRI PCa radiomics. Even with extensive experimentation, the effects of various preprocessing on improving model generalization remain topic of research. For example, Castillo et al. [25] implemented pre-processing for their radiomics MRI PCa model that did not lead to better performance (AUC single center model 0.75 vs. AUC multi-center model 0.75). All these techniques aim to improve generalization on multicenter data. Our study focuses on another aspect, namely the limitations of poor generalization with single-center data. Extending to multi-center data we show is better, but optimizing in multi-center data requires further radiomics technology research. Third, due to the use of different biopsy techniques there was a non-uniform gold standard employed as labels. Due to extensive multi-center data collection and wanting to include as much data as possible, this is almost unavoidable for bigger sets, but should be acknowledged as a limitation, nonetheless.

In conclusion, a ScSv trained radiomics-based bpMRI model does not generalize to McMv data. McMv trained radiomics-based bpMRI models do generalize, have equal single-center performance and perform better on multi-center data.

## Supplementary Information

**Additional file 1.** Detailed MR scanner information and a placement example of an auto-fixed volume of interest.

## Data Availability

The single-center PROSTATEx dataset used during the current study is available from the cancer imaging archive, https://wiki.cancerimagingarchive.net/display/Public/SPIE-AAPM-NCI+PROSTATEx+Challenges. The multi-center dataset used for this study is not publicly available due to it being a collaboration between multiple centers, having only IRB approval for this study and several agreements made for use and sharing of the data.

## Abbreviations

AI: Artificial intelligence
AUC: Area under the curve
bpMRI: Biparametric magnetic resonance imaging
CI: Confidence interval
CS: Clinically significant
DWI: Diffusion-weighted imaging
ISUP: International Society of Urological Pathology
McMv: Multi-center, multi-vendor
mpMRI: Multiparametric magnetic resonance imaging
PCa: Prostate cancer
PI-RADS: Prostate imaging-reporting and data system
PZ: Peripheral zone
ROC: Receiver operating curve
ScSv: Single-center, single-vendor
TRUS: Transrectal ultrasound
TZ: Transition zone
VOI: Volume of interest
