# Supplementary material for: Single-center versus multi-center biparametric MRI radiomics approach for clinically significant peripheral zone prostate cancer
Source: Insights Imaging. 2021 Oct 21;12:150. doi: 10.1186/s13244-021-01099-y (PMC8531183; doi:10.1186/s13244-021-01099-y)
Supplement: Supplementary file 1 — Additional file 1. Detailed MR scanner information and a placement example of an auto-fixed volume of interest. [file 13244_2021_1099_MOESM1_ESM.docx]

**ELECTRONIC SUPPLEMENTARY MATERIAL**

**Electronic supplementary material 1. Name and scanner details dataset.**

| **Name** | **Vendor** | **Scanners** | **Lesions** |
| --- | --- | --- | --- |
| Hospital A | Philips | Ingenia 3T, Achieva 1.5T, Intera 1.5T | 157 |
| Hospital B | SIemens | Skyra 3T, Prisma 3T, Aera 1.5T | 79 |
| Hospital C | Siemens | Avanto 1.5T | 44 |
| Hospital D | Philips | Achieva 1.5T | 7 |
| Hospital E | SIemens | Avanto 1.5T | 10 |
| Hospital F | SIemens | Aera 1.5T | 8 |
| Hospital G | Philips | Ingenia 3T, Achieva 1.5T | 15 |
| Hospital H | SIemens | Espree 1.5T | 7 |
| Hospital I | Siemens | Espree 1.5T | 8 |

**Electronic supplementary material 2. MRI sequence summary**

| **Sequence** | **T2-weighted imaging** | **Diffusion-weighted imaging** |
| --- | --- | --- |
| In-plane resolution range (mm) | 0.23 – 0.78 | 0.85 – 2.19 |
| Slice thickness range (mm) | 3 – 5 | 3 – 5 |
| Sequence orientation | Axial, sagittal, and coronal | Axial |
| Remarks | No endorectal coil | No endorectal coil  b-value of 800 s/mm^2^  Used for calculated b-value of > 1400 s/mm^2^ and mono-exponentially calculated apparent diffusion coefficient map |

**Electronic supplementary material 3. Auto-fixed VOI example.**

**
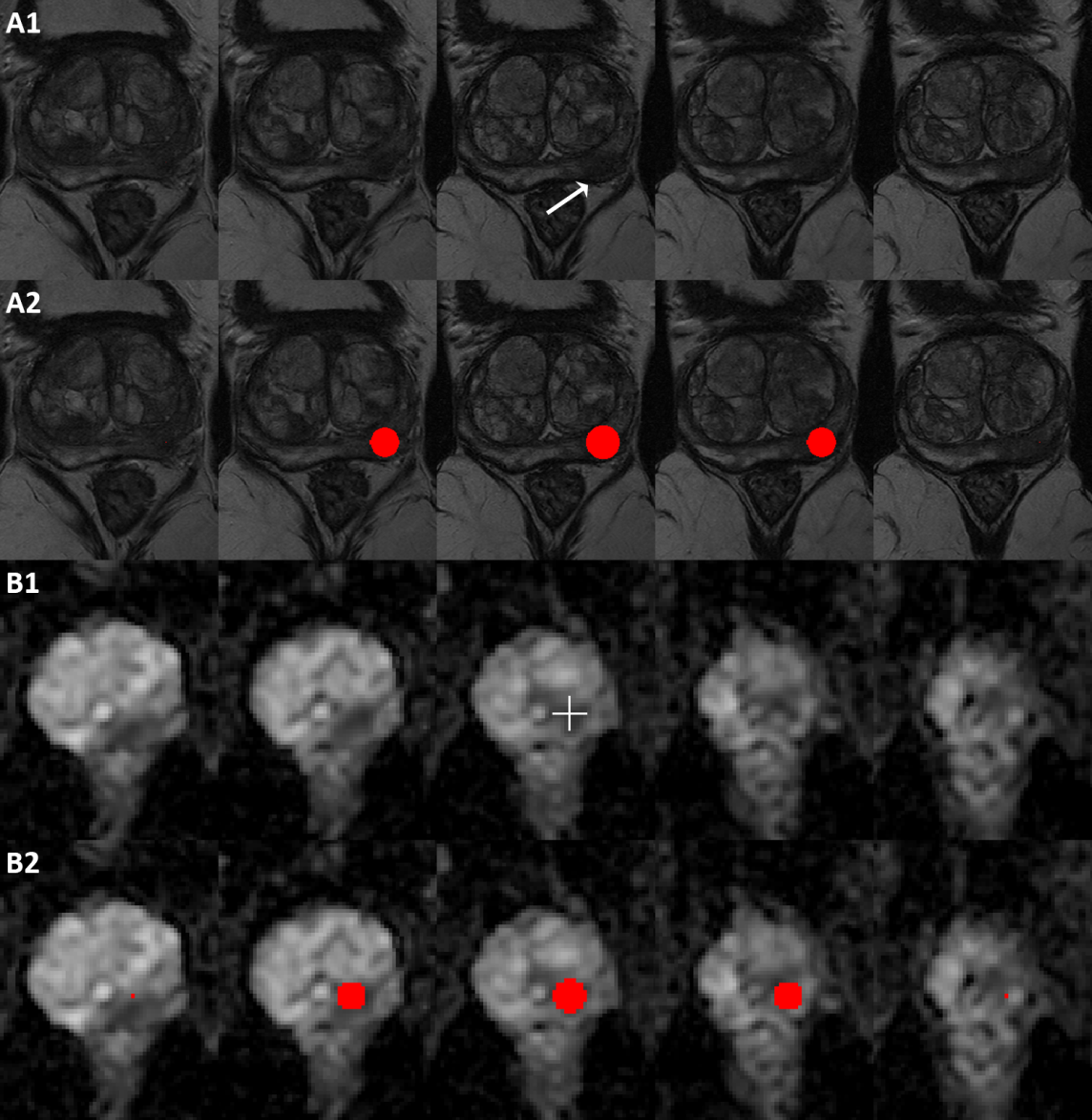
**

A1. Axial T2-weighted images of a 62-year-old man with an arrow indicating the lesion location. MRI-TRUS fusion biopsy diagnosed this as an ISUP 3 lesion. A2. 12-mm auto-fixed VOI placement on axial T2-weighted images, constructed around the apparent diffusion coefficient pinpoint. B1. Apparent diffusion coefficient map of the same patient with a mark indicating the observer placed pinpoint. B2 12mm auto-fixed VOI placement on the apparent diffusion coefficient map.
